# Upper-Extremity Dual-Task Function: An Innovative Method to Assess Cognitive Impairment in Older Adults

**DOI:** 10.3389/fnagi.2016.00167

**Published:** 2016-07-07

**Authors:** Nima Toosizadeh, Bijan Najafi, Eric M. Reiman, Reine M. Mager, Jaimeson K. Veldhuizen, Kathy O’Connor, Edward Zamrini, Jane Mohler

**Affiliations:** ^1^Arizona Center on Aging, Department of Medicine, College of Medicine, University of Arizona, TucsonAZ, USA; ^2^Interdisciplinary Consortium on Advanced Motion Performance, Department of Surgery, College of Medicine, University of Arizona, TucsonAZ, USA; ^3^Department of Neurology, College of Medicine, University of Arizona, TucsonAZ, USA; ^4^Department of Biomedical Engineering, University of Arizona, TucsonAZ, USA; ^5^Banner Sun Health Research Institute, Sun CityAZ, USA; ^6^Banner Alzheimer’s Institute, University of Arizona, TucsonAZ, USA; ^7^Department of Neurology, University of Utah, Salt Lake CityUT, USA

**Keywords:** dual-task cost, mild cognitive impairment, Alzheimer’s disease, upper-limb movements, cognition

## Abstract

**Background:** Difficulties in orchestrating simultaneous tasks (i.e., dual-tasking) have been associated with cognitive impairments in older adults. Gait tests have been commonly used as the motor task component for dual-task assessments; however, many older adults have mobility impairments or there is a lack of space in busy clinical settings. We assessed an upper-extremity function (UEF) test as an alternative motor task to study the dual-task motor performance in older adults.

**Methods:** Older adults (≥65 years) were recruited, and cognitive ability was measured using the Montreal cognitive assessment (MoCA). Participants performed repetitive elbow flexion with their maximum pace, once single-task, and once while counting backward by one (dual-task). Single- and dual-task gait tests were also performed with normal speed. Three-dimensional kinematics was measured both from upper-extremity and lower-extremity using wearable sensors to determine UEF and gait parameters. Parameters were compared between the cognitively impaired and healthy groups using analysis of variance tests, while controlling for age, gender, and body mass index (BMI). Correlations between UEF and gait parameters for dual-task and dual-task cost were assessed using linear regression models.

**Results:** Sixty-seven older adults were recruited (age = 83 ± 10 years). Based on MoCA, 10 (15%) were cognitively impaired. While no significant differences were observed in the single-task condition, within the dual-task condition, the cognitively impaired group showed significantly less arm flexion speed (62%, *d* = 1.51, *p* = 0.02) and range of motion (27%, *d* = 0.93, *p* = 0.04), and higher speed variability (88%, *d* = 1.82, *p* < 0.0001) compared to the cognitively intact group, when adjusted with age, gender, and BMI. Significant correlations were observed between UEF speed parameters and gait stride velocity for dual-task condition (*r* = 0.55, *p* < 0.0001) and dual-task cost (*r* = 0.28, *p* = 0.03).

**Conclusion:** We introduced a novel test for assessing dual-task performance in older adults that lasts 20 s and is based on upper-extremity function. Our results confirm significant associations between upper-extremity speed, range of motion, and speed variability with both the MoCA score and the gait performance within the dual-task condition.

## Introduction

Difficulties in orchestrating simultaneous tasks have been associated with cognitive impairments in older adults (Persad et al., 2008; Montero-Odasso et al., 2012). Specifically, during dual-task conditions impairments in gait and balance performance have been reported, which have been associated to deficits in attentional resources (Hauer et al., 2003; Beauchet et al., 2005; Montero-Odasso et al., 2012; Kearney et al., 2013; Schoene et al., 2014). In older adults, specially, deficits in dual-tasking are more pronounced; compared to younger individuals, older adults show higher reductions in both motor and cognitive task performances, with a higher tendency to protect the motor task in the expense of the cognitive task (Schaefer and Schumacher, 2010). Furthermore, previous studies demonstrated association between gait dual-task performance and cognitive tests (the MoCA and the MMSE; Montero-Odasso et al., 2012; Ansai et al., 2016). Poor dual-task gait performance has also been significantly associated with pathologically decreased executive and neuropsychological function, and demonstrated to be predictive of Alzheimer’s disease or even mild cognitive impairment (Camicioli et al., 1997; Sheridan et al., 2003; Montero-Odasso et al., 2012). Previous work have suggested different theoretical models to explain the nature of dual-tasking based on psychological evidence including capacity sharing (i.e., shared processing capacity or mental resources among tasks), bottlenecks (i.e., dual tasks need the same mechanism/resources at the same time), and cross-talk (i.e., interference in dual-tasking due to similarities in the content of information being processed) (Pashler, 1994).

Assessing dual-task performance is also clinically important because it represents the real life condition for performing daily activities, and may better describe the potential for adverse events such as falling (Hausdorff et al., 2005; Montero-Odasso et al., 2012; Kearney et al., 2013). Gait tests have been commonly used as the motor task component for dual-task assessments; however, many older adults have mobility impairments and high risk of falling during testing, or there is a lack of time and space in busy clinical settings, which can influence the execution of the gait test. Additionally, pencil and paper screening for cognition, is stressful and challenging for patients, and may be difficult to perform in a noisy clinical setting. Accordingly, more than half of patients with dementia never receive an evaluation of diagnosis, and a quick and objective sensitive and specific measure of cognition would be useful in clinical research settings, reducing observer bias, and decreasing subject stress. An alternative motor task assessment for dual-tasking may be beneficial from both clinical and research viewpoints for assessing cognitive impairments in older adults.

We have previously developed and validated an UEF test to assess slowness, weakness, exhaustion, and flexibility within an upper-extremity motion (Toosizadeh et al., 2015b, 2016). The UEF test integrates low-cost sensors and data acquisition system (as low as $200), the physical assessment (including preparation/calibration) is easily performed in less than 1 min, and the post-processing is performed in less than 2 min. This test was specifically validated based on the Fried index (Fried et al., 2001) to identify frailty level (non-frail, pre-frail, and frail categories) among community dwelling older adults. Additionally, in previous work we determined strong correlations between upper-extremity motion and gait speed (Toosizadeh et al., 2015b; Gary et al., 2016) and 6-min walk distance (unpublished data).

The purpose of the current study was, therefore, to assess the UEF as an alternative motor task instead of walking (gait assessment), to assess dual-task motor performance in older adults. We, specifically, examined: (1) associations between the UEF dual-task performance and validated dementia rating scales, including MoCA and MMSE; and (2) associations between UEF and gait dual-task performances. Based on our previous observations of strong associations between UEF and gait performances within single-task conditions (Toosizadeh et al., 2015b; Gary et al., 2016), we hypothesized that, similar to gait dual-task, UEF dual-task performance would decrease with the cognitive level as measured by MoCA (or MMSE), and significant correlations would exist between dual-task and dual-task cost measures within the UEF and gait tests.

## Materials and Methods

### Participants

Older adult (≥65 years) were recruited from Banner Sun Health Research Institute’s, Longevity Cohort from June 2015 to August 2015. Participants were excluded if they had known disorders associated with severe gait deficits (including stroke, diagnosed Parkinson’s disease, recent surgery, major foot deformity such as Charcot neuroarthropathy, or foot amputation). Furthermore, participants with major mobility disorders (e.g., who were unable to walk a distance of 20 m without walking assistance), and upper-extremity disorders (including severe shoulder or elbow osteoarthritis) were also excluded. Assistive devices, used routinely by participants in their daily activities, including canes and walkers, but excluding wheelchairs, were allowed. The study was approved by the University of Arizona’s and Banner Sun Health Research Institute’s Institutional Review Boards. Written informed consent according to the principles expressed in the Declaration of Helsinki (World Medical Association, 2013) was obtained from all subjects (or an authorized person in case of lack of clinically assessed capacity for informed consent) before participation.

### Clinical Measurements

Clinical measurements included MoCA (Nasreddine et al., 2005), MMSE (Folstein et al., 1975), the CES-D scale (Orme et al., 1986), and the Fried frailty index (Fried et al., 2001). Both MoCA and MMSE were used, separately, as standard methods for assessing cognitive impairments. Participants with MMSE score less than 24 were identified as those with moderate to severe cognitive impairments (Tombaugh and McIntyre, 1992). MoCA was used as a more sensitive test for identifying participants with mild cognitive impairment; a cutoff of 20 ≥ 30 was used for MoCA to indicate cognitively intact status as has been recently suggested to provide better accuracy (compared to original cutoff of 26 ≥ 30) in those with unselected cognitive disorders (Hancock and Larner, 2011; Larner, 2012). Both MMSE and MoCA scores were adjusted with normative age and education level based on previous work (Measso et al., 1993; Malek-Ahmadi et al., 2015). The CES-D short version scale was used to measure self-reported depression symptoms. Fried index [unintentional weight loss, self-reported exhaustion, weakness (grip strength), slow gait speed (15 feet gait test), and self-reported low physical activity] was used to assess frailty (Fried et al., 2001). Individuals with one or two positive Fried criteria were considered pre-frail and those with three or more, frail, and those with none, non-frail.

### UEF Measurements

**FIGURE 1:**
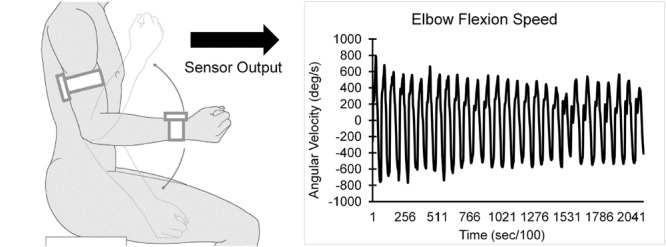
**UEF experimental setup and sensor output (i.e., elbow angular velocity)**.

Each participant performed a 20-s trial of elbow flexion, within which they repetitively flexed and extended their dominant elbow to full flexion and extension as quickly as possible in seated position, while wearing the UEF system. Before the actual test, participants performed a short practice trial on their non-dominant arm to become familiar with the protocol. The protocol was explained to participants and they were encouraged only once, before the elbow flexion, to do the test as fast as possible (participants were not further encouraged during the test). A tri-axial wearable gyroscope and accelerometer sensor (sample frequency = 100Hz, BioSensics LLC, Boston, MA, USA), was attached to the upper-arm near the biceps and one to the wrist, both on the dominant arm, using a band attached with Velcro, to estimate three-dimensional angular velocity of the upper-arm and forearm segments, and ultimately elbow flexion (**Figure 1**).

Several outcome measures representing kinematics and kinetics of the elbow flexion were derived using angular velocity and anthropometric data (i.e., participants’ gender, stature, and body mass). UEF outcome measures included: (1) speed; (2) flexibility; (3) power; (4) rise time; (5) moment; (6) speed reduction; (7) speed variability; and (8) flexion number (see **Table 1** for definitions). Speed, rise time, and flexion number were used to assess slowness of movement, power and moment were used to assess acceleration and weakness, and exhaustion was estimated using speed reduction data within 20 s of elbow flexion. Furthermore, speed variability was measured here as the speed coefficient of variation (CoV = SD of speed divided by mean speed) within 20 s of elbow flexion. Speed variability was measured here to represent motor performance variability that can potentially be influenced by performing a dual-task, because previous studies demonstrated a larger effect of dual-task on gait variability (gait speed CoV) compared to gait speed itself (Beauchet et al., 2005; Montero-Odasso et al., 2012) (Readers are referred to (Toosizadeh et al., 2015b) for more details regarding validation of UEF using a reference motion capture system, and detailed description of parameter calculations.)

**Table 1:** UEF and gait parameter definitions.

	Definition	Reference
**UEF Parameters**		
Speed	Mean value of elbow angular velocity range (maximum minus minimum speed)	Toosizadeh et al., 2015b
Flexibility	Mean value of elbow flexion range	Toosizadeh et al., 2015b
Power	Mean value of product of the angular acceleration range and the range of angular velocity	Toosizadeh et al., 2015b
Rise time	Mean value of the required time to reach the maximum angular velocity	Toosizadeh et al., 2015b
Moment	Mean value of maximum moments on elbow within each flexion/extension estimated from moment of inertia of forearm and hand, and elbow motion	Toosizadeh et al., 2015b
Speed reduction	Difference in angular velocity range between the last and the first 5 s of elbow flexion as a percentage of the initial angular velocity range	Toosizadeh et al., 2015b
Speed variability	CoV of the angular velocity range during 20 s	Toosizadeh et al., 2015b
Flexion number	Number of flexion/extensions during 20 s	Toosizadeh et al., 2015b
**Gait Parameters**		
Stride velocity	Average of gait speed (horizontal distance traveled divided by duration of walking) among strides	Aminian et al., 2002
Stride time	Time interval starts when one foot makes contact with the ground and ends when that same foot contacts the ground again	Aminian et al., 2002
Stride length	Distance traveled by the same limb between two successive heel contacts	Aminian et al., 2002
Double support	Duration of the initial and terminal double support when both feet are in contact with the ground as a percentage of the stride time	Aminian et al., 2002
Gait variability	CoV of gait speed among strides	Aminian et al., 2002

*A reference for calculation procedure is presented for each parameter. CoV, coefficient of variation (standard deviation divided by the mean).*

### Gait Measurements

Gait was objectively assessed using validated wearable technology. Three-dimensional acceleration and angular velocity of shins were measured using two wearable sensors (the same sensors used in UEF measurements), each of which included a tri-axial gyroscope, and a tri-axial accelerometer (sample frequency = 100 Hz, BioSensics, Boston, MA, USA), to derive gait outcome measures using established methods previously reported (Aminian et al., 2002; Najafi et al., 2009, 2010). Sensors were attached to the shins above the ankle. Gait was assessed within a minimum of 25 steps at a normal desired pace that participants perform everyday activities. Gait outcome measures included: (1) stride velocity; (2) stride time; (3) stride length; (4) double support; and (5) gait variability (see **Table 1** for definitions). All parameters were measured during steady state of walking, by removing gait initiation and termination using an algorithm described in our previous work (Lindemann et al., 2008; Toosizadeh et al., 2015a).

### Dual-Task Protocol and Dual-Task Cost Measurements

Both UEF and gait tests were followed immediately by a dual-task condition test, within which participants were asked to maintain their usual speed for the test (i.e., as fast as possible for UEF and normal desired speed for gait), while counting numbers backward by one at their self-selected rhythm. Counting numbers was selected here as the dual-task because it involves working memory (Lee and Kang, 2002), and, therefore, is more directly related to executive functions, compared to other tasks such as naming objects/animals (Smith et al., 2001; Beauchet et al., 2005; Montero-Odasso et al., 2012). Also, counting is a rhythmic task and may highly interfere with another rhythmic task that has a different frequency such as walking or repetitive elbow flexion (Taga et al., 1991; Beauchet et al., 2005). The dual-task of counting backward by “one” was chosen here, since it has been proven to be simpler and more appropriate for older adults (Beauchet et al., 2003). To better represent the natural environment in performing daily activities, there was no instruction to prioritize either the physical task or the counting task (Hittmair-Delazer et al., 1994; Weiss et al., 2003; Montero-Odasso et al., 2012).

Each of the above UEF and gait parameters were measured within both single-task (no counting), and dual-task (counting) conditions. To assess changes in individual’s performance from a single to a dual-task, dual-task “cost” was measured for each parameter as percentage of change within two conditions:

(1) $${\mathrm{Parameter}}_{dual-task\;\mathrm{cost}}=\frac{{\mathrm{Parameter}}_{dual-task}{-Parameter}_{single-task}}{{\mathrm{Parameter}}_{single-task}}$$

Of note, to assure that a higher positive percentage value of dual-task cost represents deteriorated motor performance for all parameters, the dual-task cost values were multiplied by -1 for rise-time, speed reduction, and speed variability UEF parameters, and stride time, double support, and gait variability gait parameters.

To assess the secondary task performance (i.e., counting numbers), the number of correctly counted numbers within the 20-s arm test was considered as an outcome. For this purpose, the number of mistakes was subtracted from the total counted numbers to determine the correctly counted numbers. This parameter represents the speed and accuracy of the secondary task within the dual-task condition (Hauer et al., 2003).

### Statistical Analysis

UEF parameters (single-task, dual-task, and dual-task cost) were compared between two groups of cognitively intact and cognitively impaired participants (defined by MoCA) using separate analyses of variance (ANOVAs); age, gender, and BMI were considered as covariates and Cohen’s effect size (d) was estimated. Correlations between UEF parameters and the MoCA score (and MoCA sub-categories including visuospatial/executive, naming, attention, language, abstraction, delayed recall, and orientation) were assessed using linear multiple regression-ANOVA models with each UEF parameter, age, gender, and BMI as independent variables and the MoCA score (or the MoCA subcategory score) as the dependent variable. Further, a multiple logistic regression model was used to determine the association between UEF parameters combined and cognition status. In this model, UEF parameters and demographic parameters with significant ANOVA between group differences (*p* < 0.01) among cognition groups were considered as independent variables, and two groups of cognitively intact and cognitively impaired (defined by MoCA) was considered as the dependent variable; sensitivity, specificity, and area under curve (AUC) were estimated using receiver operating characteristic (ROC) curves for identifying cognitively impaired participants. Furthermore, to compare UEF and gait within the dual-task condition, correlations between UEF and gait dual-task and dual-task cost parameters were assessed using linear simple regression-ANOVA models. For all correlations linear Pearson values (r) were reported. ANOVA or χ^2^ tests (as relevant) were performed to evaluate the differences in demographic and clinical parameters between two groups of cognitively intact and cognitively impaired participants (defined by MoCA). A summary of results is presented as mean (SD). All analyses were done using JMP (Version 11, SAS Institute Inc., Cary, NC, USA), and statistical significance was concluded when *p* < 0.05.

## Results

### Participants

Seventy three older adults were screened for the study, among which 67 (92%) were able to complete all physical tests. Six individuals (8%) were not eligible for the study due to an inability to perform either the UEF or the gait test. Among participants, 20 (30%) were male, with a mean (SD) age and BMI of 82 (11) years and 25.66 (4.09) kg/m^2^, respectively; corresponding values were 83 (10) years and 25.69 (4.52) kg/m^2^ for females. Respectively, 10 (15%) and 3(4%) of participants were diagnosed with cognitive impairment using MoCA and MMSE tests. As expected, cognitively impaired participants (defined by MoCA) were significantly older than cognitively intact individuals (*p* < 0.001). The percentage of pre-frail/frail individuals was also higher among the cognitively impaired group; however, the difference was not significant (*p* = 0.07). Depression score was not significantly different between two groups of cognitively intact and cognitively impaired participants (*p* = 0.58). Further, none of participants had problem understanding questions and counting numbers in English as all of them were English speakers. All socio-demographic data and clinical information are reported in **Table 2**.

**Table 2:** Sociodemographic and clinical measures.

Variable	Male	Female	Total	Cognitively intact (MoCA ≥ 20)	Cognitively impaired (MoCA < 20)	*p*-value^†^
Number (% of the group)	20 (30%)	47 (70%)	67	57 (85%)	10 (15%)	–
Age, year (SD)	82 (11)	83 (10)	83 (10)	81 (10)	93 (5)	<0.001^∗^
Stature, cm (SD)	173.93 (8.10)	160.22 (6.59)	164.31 (9.44)	164.26 (9.49)	164.70 (9.65)	0.90
Body mass, kg (SD)	77.74 (14.17)	66.21 (12.93)	69.65 (14.23)	70.56 (14.71)	62.95 (7.60)	0.16
BMI, kg/m^2^ (SD)	25.66 (4.09)	25.69 (4.52)	25.68 (4.38)	26.02 (4.51)	23.19 (1.78)	0.07
Education level						
Primary, n (% of the group)	0 (0)	0 (0)	0 (0)	0 (0)	0 (0)	–
Secondary, n (% of the group)	1 (5%)	7 (15%)	8 (12%)	9 (16%)	1 (10%)	0.91
High school, n (% of the group)	19 (95%)	40 (85%)	59 (88%)	48 (84%)	9 (90%)	0.91
MoCA score, 0–30 (SD)	24.45 (3.19)	24.13 (4.41)	24.23 (4.05)	25.23 (2.94)	16.50 (2.98)	–
MMSE score, 0–30 (SD)	26.53 (1.90)	27.16 (2.07)	26.97 (2.02)	27.45 (1.39)	23.14 (2.27)	<0.0001^∗^
CES-D score, 0–30 (SD)	3.05 (3.63)	3.98 (4.56)	3.69 (4.29)	3.98 (4.44)	3.00 (3.51)	0.58
Pre-frail/frail, n (% of the group)	10 (50%)	30 (64%)	40 (60%)	31 (54%)	9 (90%)	0.07

*The asterisk symbol represents a significant difference. ^†^*p*-value for comparison of variables among cognition groups. MoCA, Montreal cognitive assessment; BMI, body mass index; MMSE, mini–mental state examination; CES-D, center for epidemiologic studies depression. For each gender and cognition groups the mean and standard deviation (SD) values are presented.*

### Association between UEF Dual-Task Performance with MoCA and MMSE

**FIGURE 2:**
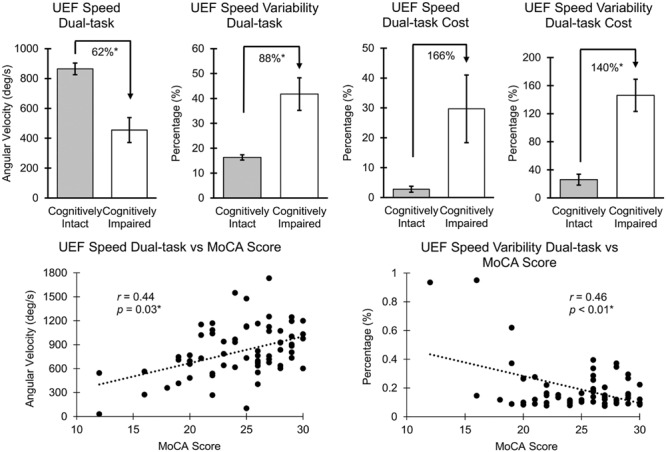
**Association between UEF speed and speed variability with MoCA.** Mean and standard error (SE) values of UEF parameters are presented, and the asterisk symbol represents a significant association. *r* values were not adjusted with age, gender, and BMI; *p*-values were adjusted with age, gender, and BMI.

Results from ANOVA demonstrated that UEF performance was, in general, worse in cognitively impaired participants (defined by MoCA) in both single- and dual-task conditions. However, when adjusting for age, gender, and BMI, only UEF parameters within the dual-task condition showed significantly worse performance in cognitively impaired participants. Specifically, speed, flexibility, and speed variability were significantly different between cognitively intact and cognitively impaired participants (**Table 3**; **Figure 2**); the largest effect size of between-group differences was observed in speed variability within the dual-task condition (*d* = 1.82, *p* < 0.0001). In contrary to the dual-task condition, none of the UEF parameters were significantly different between two groups when comparing under the single task condition (*d* < 0.76, *p* > 0.16). Results from dual-task cost showed that among UEF parameters, speed, power, flexibility, and speed variability were significantly larger (more than five times larger) in cognitively impaired compared to cognitively intact participants (*d* = 0.58–1.93, *p* < 0.03, **Table 3**). Results from the logistic regression model (using UEF and demographic parameters) showed that, cognitive impairment (defined by MoCA) was identified with sensitivity and specificity of 100 and 87% (AUC = 0.94, **Table 4**). Using age as the only predictor variable of cognition groups, sensitivity and specificity of 100 and 71% (AUC = 0.84) were achieved. According to these results adding three UEF parameters of dual-task speed variability, flexibility dual-task cost, as well as correctly counted number (secondary task performance) can improve specificity of cognitive impairment prediction by 23%.

**Table 3:** The association between MoCA and the UEF dual-task performance.

Variable	Cognitively intact (MoCA ≥ 20)	Cognitively impaired (MoCA < 20)	*p*-value^†^	*F*(4,66)^‡^	95% CI (Lower)	95% CI (Upper)	Effect size	Correlation with MoCA (*r* and *p*-value)^¶^
Speed dual-task (deg/s)	864.81 ± 299.91	455.05 ± 237.20	0.02^∗^	5.10	-274.82	-25.41	1.51	*r* = 0.48, *p* < 0.01^∗^
Flexibility dual-task (deg)	100.58 ± 29.26	73.60 ± 28.91	0.04^∗^	2.71	-25.86	-0.80	0.93	*r* = 0.37, *p* < 0.01^∗^
Power dual-task (deg^2^/s^3^ x 100000)	152.36 ± 125.07	36.53 ± 32.79	0.16	2.72	-87.62	14.60	1.27	*r* = 0.50, *p* < 0.01^∗^
Rise time dual-task (s/100)	26.05 ± 7.29	30.19 ± 4.49	0.69	3.21	-2.37	3.55	0.68	*r* = -0.22, *p* = 0.45
Moment dual-task (Nm)	1.08 ± 0.72	0.51 ± 0.38	0.37	1.35	-0.34	0.13	0.99	*r* = 0.35, *p* = 0.23
Speed reduction dual-task (%)	14.04 ± 14.39	16.63 ± 45.39	0.73	0.03	-7.40	10.53	0.08	*r* = -0.12, *p* = 0.27
Speed variability dual-task (%)	16.30 ± 8.24	41.75 ± 18.52	< 0.0001^∗^	3.29	7.29	20.00	1.82	*r* = -0.50, *p* < 0.01^∗^
Flexion number dual-task	23.95 ± 6.52	17.50 ± 3.16	0.32	9.31	-3.47	1.17	1.26	*r* = 0.37, *p* = 0.58
Speed dual-task cost (%)	2.77 ± 57.64	29.68 ± 32.02	0.01^∗^	2.37	-8.99	39.75	0.58	*r* = -0.21, *p* = 0.05
Flexibility dual-task cost (%)	0.73 ± 22.43	12.92 ± 16.91	< 0.01^∗^	2.41	-0.80	18.97	0.61	*r* = -0.22, *p* = 0.04^∗^
Power dual-task cost (%)	9.25 ± 52.83	46.36 ± 29.87	0.01^∗^	2.13	2.40	46.70	0.86	*r* = -0.31, *p* < 0.01^∗^
Rise time dual-task cost (%)	13.15 ± 27.37	11.69 ± 35.62	0.13	0.76	-13.37	13.58	0.05	*r* = -0.16, *p* = 0.19
Moment dual-task cost (%)	12.10 ± 21.16	19.07 ± 20.29	0.36	0.94	-4.91	13.39	0.34	*r* = -0.09, *p* = 0.37
Speed reduction dual-task cost (%)	0.20 ± 5.31	2.33 ± 3.97	0.31	0.42	-3.53	1.13	0.48	*r* = -0.10, *p* = 0.60
Speed variability dual-task cost (%)	25.95 ± 59.80	146.14 ± 64.94	0.03^∗^	2.76	3.84	103.62	1.93	*r* = -0.25, *p* = 0.08
Flexion number dual-task cost (%)	8.92 ± 14.60	20.17 ± 22.66	0.22	1.78	-3.19	13.62	0.62	*r* = -0.29, *p* = 0.05
Correctly counted numbers	24.34 ± 7.39	15.63 ± 16.75	< 0.01^∗^	3.08	-9.73	-2.09	0.67	*r* = 0.27, *p* = 0.18

*For each group of cognitively intact and cognitively impaired participants, mean and standard deviation (SD) values of UEF parameters are presented. Correlations between UEF parameters and MoCA scores are presented as linear Pearson (*r*) values. Results are presented only for the dual-task condition and dual-task cost, since no significant association between cognitive impairment and UEF parameters was determined within the single-task condition. The asterisk symbol represents a significant association. ^†^ANOVA *p*-value for UEF parameter differences between cognition groups, adjusted with age, gender, and body mass index (BMI). ^‡^*F*-values for each UEF parameter as the dependent variable and cognition groups, age, gender, and BMI as independent variables. Numerator degrees of freedom for the *F* ratio = 4; denominator degrees of freedom for the F ratio = 66. ^¶^For r values Pearson correlations between each UEF parameter and the MoCA score are reported using simple regression unadjusted with age, gender, and BMI. For *p*-values Pearson correlations between each UEF parameter and the MoCA score are reported using multiple regressions adjusted with age, gender, and BMI.*

*MoCA, Montreal cognitive assessment; CI, confidence interval.*

**Table 4:** Results of the multiple logistic regression model.

Independent variables	Parameter estimates	Standard errors	χ^2^	*p*-value	95% CI (Lower)	95% CI (Upper)
Intercept	19.58	8.28	5.60	0.02^∗^	5.92	40.04
Age (year)	-0.04	0.09	0.25	0.62	-0.23	0.13
Speed variability dual-task (%)	-5.33	4.22	1.59	0.21	-15.31	2.28
Flexibility dual-task cost (%)	-3.86	5.61	0.47	0.49	-17.48	6.01
Correctly counted numbers	-0.38	0.25	2.28	0.13	-1.06	-0.04

*Dependent variable: cognition groups defined by MoCA; independent variables: UEF parameters and demographic parameters (with significant ANOVA between group differences (*p* < 0.01) among cognition groups). The asterisk symbol represents a significant independent association. CI, confidence interval.*

Several UEF parameters were correlated with the MoCA score within both single- and dual-task conditions; however, when age, gender, and BMI were included in the regression model, only speed, flexibility, power, and speed variability parameters within the dual-task condition and dual-task cost for flexibility and power were significantly (and independent of demographic data) correlated with the MoCA score (**Table 3**; **Figure 2**). Speed variability within the dual-task condition demonstrated the largest Pearson correlation with the MoCA score (*r* = 0.50); the lower the MoCA score, the higher UEF speed variability while counting numbers. Of note, a significant negative Pearson correlation was also observed between age and the MoCA score (*r* = -0.54, *p* < 0.001). When dual-task speed variability and age considered as independent variables together, and the MoCA score as the dependent variable, the regression Pearson correlation improved to *r* = 0.64. Furthermore, comparing separate associations of age and MoCA with UEF dual-task speed variability, showed a better correlation between MoCA and UEF performance (*r* = -0.50, *p* < 0.01) compared to age and UEF performance (*r* = 0.24, *p* = 0.03). Also, in a regression model with UEF dual-task speed variability as the dependent variable, and both the MoCA score and age as independent variables, only the MoCA score showed significant independent association with the UEF performance (*p* < 0.01) but not age (*p* = 0.41). None of the UEF parameters were significantly associated with MoCA score within the single-task condition when adjusted with age, gender, and BMI (*r* < 0.24, *p* > 0.10).

The secondary task measure, correctly counted numbers, was significantly larger among cognitively intact participants by 56% (*d* = 0.67, *p* < 0.01, **Table 3**). Furthermore, although the correctly counted numbers was positively correlated with the MoCA score, the association was not significant (**Table 3**).

Further, among UEF parameters, significant positive correlations were observed between dual-task speed with visuospatial/executive, attention, delayed recall, and orientation MoCA subcategories (*r* = 0.29–0.33, *p* < 0.05). The secondary task of correctly counted numbers was also demonstrated significant positive correlations with the language MoCA subcategory (*r* = 0.41, *p* < 0.001).

### Association between UEF and Gait Dual-Task Performances

Within the dual-task condition, the strongest correlations were observed between UEF speed and gait stride velocity (*r* = 0.55, *p* < 0.0001), UEF flexibility and gait stride velocity (*r* = 0.32, *p* < 0.01), UEF power and gait stride velocity (*r* = 0.54, *p* < 0.0001), UEF rise time and gait stride velocity (*r* = 0.30, *p* = 0.02), UEF moment and gait stride length (*r* = 0.59, *p* < 0.0001), and UEF flexion number and gait stride length (*r* = 0.50, *p* < 0.0001). No significant correlation was found between UEF speed reduction and speed variability with any of the gait parameters within the dual-task condition (*r* < 0.28, *p* > 0.06). Overall, significant correlations, suggest an association between upper- and lower-extremity motions within the dual-task condition. Of note, several other associations exist between UEF and gait parameters; however, only those with the strongest correlation for each of UEF parameter are presented.

Comparing the dual-task cost in performing UEF and gait tests revealed significant correlations only between UEF rise time and gait stride velocity (*r* = 0.28, *p* = 0.03), UEF moment and gait double support (*r* = 0.26, *p* = 0.03), and UEF flexion number and gait stride length (*r* = 0.33, *p* < 0.01).

## Discussion

### Assessing Cognitive Impairment using Upper-Extremity Dual-Task Performance

As we hypothesized, older adults with lower cognitive scores showed worse UEF performance within the dual-task condition. We also observed a reduction in UEF performance with increase in MoCA score within the single-task condition, since as people get older simultaneous declines in motor function and cognitive function, especially attention and memory, occur, as has been reported in previous research (Volkow et al., 1998; Verhaeghen et al., 2003; Blazer et al., 2015). A significant negative correlation here between the MoCA score and age confirms this fact. Interestingly, when statistical analyses were adjusted for age (and gender and BMI), no significant association was observed between UEF performance and the MoCA score within the single-task condition. On the other hand, there were significant associations between UEF dual-task parameters and dual-task cost with MoCA, even when adjusting for age, suggesting that performing a dual-task of counting numbers backward could amplify the cognitive impairment in older adults.

Among UEF parameters only those related to agility, flexi bility, and accuracy (variability) of performance were different between cognitively intact and cognitively impaired participants. As reported in **Table 3**, speed, power (representing acceleration), and range of elbow flexion (representing flexibility), as well as speed variability during elbow flexion were worse in the cognitively impaired group. While, UEF parameters that are more related to strength (elbow moment) or exhaustion (speed reduction) showed no significant difference between two groups. This is in agreement with previous research based on gait performance, within which, gait variability and stride velocity within the dual-task condition have been reported as the most sensitive parameters for differentiating cognitively impaired from healthy participants, compared to other spatial-temporal gait parameters (Beauchet et al., 2005; Al-Yahya et al., 2011; Montero-Odasso et al., 2012; Muir et al., 2012).

Within our sample, we observed that visuospatial/executive, attention, delayed recall, and orientation MoCA subcategories are better correlated with the UEF dual-task performance. Previous work has associated the delayed recall and orientation components of the MoCA test to memory deficits, based on domain-specific detailed conventional neuropsychological testing (Lam et al., 2013). Therefore, although associations are moderate, findings here suggest that UEF dual-task can better identify working memory and executive functioning deficits in older adults, compared to impairments in other cognitive domains. Montero-Odasso et al. (2009) has reported similar results within dual-task walking performance measures; they reported that working memory and executive function performances measured, respectively, with the Letter Number Sequencing Test (Wechsler et al., 2008) and the Trail Making Test (Corrigan and Hinkeldey, 1987; Tombaugh, 2004) are associated with slower walking within dual-task conditions. Interestingly, a significant association was also observed between the attention subcategory of MoCA with the UEF dual-task speed; however, the association was weak. Weaker association between attention measures and dual-task walking deficits (compared to executive function and working memory domains) was also reported previously (Montero-Odasso et al., 2009). Although, the current findings may confirm previously reported insights regarding the interactions between cognitive domains and dual-task performance, future investigations are required using more domain-specific neuropsychological tests to provide robust conclusions regarding attention, working memory, and executive functioning decline and compromised dual-task performance.

Over the past decade, several researchers have focused on investigating motor dysfunction due to cognitive impairments, specifically, studying impairments in brain control of gait within a dual-task condition. Counting numbers backward, which is introduced as a mental tracking task, is suggested to share similar neural network in the brain that are interlinked with those of gait control, and therefore, can disturb gait performance (Fuster, 2003; Gazzaley and D’Esposito, 2006; Al-Yahya et al., 2011). We previously, reported a strong correlation between rapid upper-extremity motion and gait speed within a single-task condition (Toosizadeh et al., 2015b), and here, we observed that correlations exist between upper- and lower-extremity motions within the dual-task condition. The better people execute walking, the better they execute the rapid elbow flexion task. Although rapid elbow flexion seems to be an easy task to perform, it requires a complex neuromuscular control to accelerate and decelerate the upper-arm and the forearm movements with an optimum timing of agonist and antagonist muscle activations, while maintaining dynamic elbow stability (Raikova and Aladjov, 2003; Raikova et al., 2005; Li and Kuanyi, 2007).

### Clinical Implications and Future Directions

A quick and objective, sensitive and specific screening measure of cognition would be useful for research and in clinical settings, reducing observer bias, and decreasing subject stress. The reported method may serve as a useful future cognition screening based on dual-task modality, but may lack the precision necessary as a diagnostic tool or for tracking cognition over time. Although gait dual-task testing provides high reliability for measuring cognitive impairment in older adults, performing this test is often clinically burdensome, and unsuitable for bedbound individuals and patients with mobility limitations. Results from the current study, suggest that an alternative motor task performance, such as a simple rapid elbow flexion while performing a secondary task, to some extent, may provide valuable information about cognitive status of older adults. Accordingly, UEF test with a self-selected speed, as observed in our pilot results, may not be attention-demanding enough. Therefore, we proposed the “rapid” UEF test to better highlight speed and accuracy components of attention based on elbow speed and speed alterations within the dual-task condition. Furthermore, several recent studies have demonstrated association between impaired dual-task gait performance and higher risk of falls in cognitively impaired older adults (Springer et al., 2006; Toulotte et al., 2006). Also, mild cognitive impairment measured using dual-task gait test has reported as a strong predictor of progression to more severe cognitive impairments and Alzheimer’s disease (Verghese et al., 2002; Waite et al., 2005). Since we observed a strong correlation between upper- and lower-extremity motions within the dual-task condition, longitudinal research exploring the association between dual-task UEF performance with fall risk and cognitive impairment progression may be beneficial.

UEF dual-task performance may also provide implications for understanding the recovery of motor performance after neurological injury, especially for stroke patients who showed up to 70% progression to dementia (Plummer-D’Amato et al., 2008; Godefroy et al., 2011). Of note, previous research suggested higher reliability of upper-extremity testing compared to lower-extremity for assessing motor performance in rehabilitation settings in stroke patients (Sanford et al., 1993; Wolf et al., 2001; Gladstone et al., 2002). Accordingly, the proposed arm test may provide additional information to study interactions between cognitive processing and motor behavior, as opposed to walking or lower-extremity function that are more commonly performed within daily activities. Our findings here suggest promise for future investigation exploring upper-extremity dual-tasking to further improve the clinical impact using other motor task performances/protocols such as key pressing or finger movements. Further, within the current approach, assessment of single-task motor performance can provides baseline data related to musculoskeletal disorders (such as arthritis) in older adults. Hence, using dual-task cost outcomes may provide more meaningful evaluation of neurological diseases by minimizing the effect of musculoskeletal disorders.

Application of dual-tasking, especially combination of motor performance and cognitive task, provides the advantage of assessing human cognition under several difficulty levels. Unlike subjective questionnaires, a dual-task difficulty can be altered by changing the cognitive component (e.g., counting backward by three or seven), or the motor task (e.g., elbow flexion vs. gait test). Our results here suggest that the motor performance component of the proposed dual-task is more associated with working memory and executive functioning, and the cognitive task performance with language. Increasing the demand for each component (motor vs. cognitive task) would, therefore, provide an additional tool for assessing syndromes to help identify type of dementia. Previous work demonstrated that type 1 dementia affects cortical part of the brain with symptoms on memory and language, such as in Alzheimer’s disease and dementia with Lewy bodies (Royall and Polk, 1998; Román and Royall, 1999; Calderon et al., 2001). On the other hand, type 2 dementia impacts frontal-subcortical, frontal, or prefrontal regions of the brain, which results in more executive dysfunctioning that is common among patients with vascular dementia or behavioral variant frontotemporal dementia (Román and Royall, 1999; Gregory et al., 2002; Hornberger et al., 2008). In future studies we will address the strength of the UEF tests for detecting syndromes related to different type of dementia in confirmation with robust neuropsychological tests.

Lastly, as mentioned above, UEF has been previously validated as a new method for assessing frailty based on physical function (Toosizadeh et al., 2015b). Clinical frailty syndrome identifies homeostenotic older adults with low physiological reserves and increased vulnerability to stressors (Fried et al., 2001; Rockwood et al., 2007). Cognitive impairments have been demonstrated to be a component of frailty, and associated with higher risk of adverse health outcomes in older adults (Rockwood et al., 2004, 2005). Accordingly, Avila-Funes et al. (2009) showed that adding a cognitive measure of frailty to physical function measures, improves the longitudinal prediction of adverse health outcomes. The current findings, therefore, suggest that adding dual-task UEF to the original protocol (20-s single-task UEF test) may enhance frailty assessment by including the cognition component of frailty. Furthermore, UEF dual-task measurement may prove sensitive to change over time. These hypotheses should be confirmed in larger prospective studies.

### Limitations

Our sample was selected from older adults living in the home and community settings, in Sun City, AZ, USA (not including long term care), who were participants in the Banner Sun Health Longevity Cohort, thus they may represent a healthier and highly educated older adult cohort than those living in other communities, who are of equivalent age.

Based on the implemented cutoff of 20 < 30 for identifying the cognitively impaired group, only 15% of participants were assigned to this group. Although the number of participants in the cognitively impaired group is limited, we still believe that our conclusions regarding the existing association between dual-task UEF and MoCA is valid, since significant correlations were observed between continuous values of dual-task UEF parameters and MoCA scores. Another limitation was the absence of clinical diagnosis and neuropsychiatric staging of cognitive impairment. Further, we acknowledge that although the effect of age as a potential confound was partly (but not fully) mitigated by statistical corrections, conclusions should be interpreted with cautious and further confirmed with more extensive cognitive tests. Although, strong association between the MoCA score and UEF performance was observed here, it should be noted that cognition assessment is a complex and multidimensional process, and therefore, no direct conclusion of association between cognitive status and UEF dual-task performance can be deducted from the current results.

As with measurement limitations in gait-based measures, upper-extremity disability or injury may limit UEF measure ments. Also, the current study lacks intra- and inter-rater reliability assessments; however, as we previously validated the UEF test among a larger sample of older adults within a different experimental settings, we observed significant correlations between all UEF parameters and gait speed (*r* = 0.38 – 0.68; *p* < 0.001) (Toosizadeh et al., 2015b).

## Conclusion

In summary, within the current study, we introduced a new test for assessing dual-task performance in older adults that lasts 20 s and is based on upper-extremity performance. This method provides the advantage of precise assessment of several aspects of motor performance (including flexibility, speed, strength, as well as exhaustion). Our results confirm strong associations between upper-extremity speed, range of motion, and speed variability with both the MoCA score and the gait performance within the dual-task condition. Current findings, although require further investigations, introduce a promising method for assessing cognition status in older adults.

## Author Contributions

NT: study design, data analysis, writing, interpretation of findings, final approval, and agreement to be accountable for all aspects of the work. BN: study design, writing, interpretation of findings, final approval, and agreement to be accountable for all aspects of the work. ER: study design, writing, interpretation of findings, final approval, and agreement to be accountable for all aspects of the work. RM: data collection, data analysis, writing, interpretation of findings, final approval, and agreement to be accountable for all aspects of the work. JV: data collection, data analysis, writing, interpretation of findings, final approval, and agreement to be accountable for all aspects of the work. KO: data collection, data analysis, writing, interpretation of findings, final approval, and agreement to be accountable for all aspects of the work. EZ: study design, writing, and interpretation of findings, final approval, and agreement to be accountable for all aspects of the work. JM: study design, writing, and interpretation of findings, final approval, and agreement to be accountable for all aspects of the work.

## Conflict of Interest Statement

The authors declare that the research was conducted in the absence of any commercial or financial relationships that could be construed as a potential conflict of interest.

## Abbreviations

ANOVA: analysis of variance
AUC: area under curve
BMI: body mass index
CES-D: center for epidemiologic studies depression
CI: confidence interval
CoV: coefficient of variation
MMSE: mini–mental state examination
MoCA: Montreal cognitive assessment
ROC: receiver operating characteristic
SD: standard deviation
SE: standard error
UEF: upper-extremity function
